# Combination of intramedullary rod, wrapping bone grafting and Ilizarov’s fixator for the treatment of Crawford type IV congenital pseudarthrosis of the tibia: mid-term follow up of 56 cases

**DOI:** 10.1186/s12891-016-1295-1

**Published:** 2016-10-22

**Authors:** Guang-hui Zhu, Hai-bo Mei, Rong-guo He, Yao-xi Liu, Kun Liu, Jin Tang, Jiang-yan Wu

**Affiliations:** Department of Orthopedics, Hunan Children’s Hospital, 86 Ziyuan road, Changsha, Hunan People’s Republic of China

**Keywords:** Congenital pseudarthrosis of tibia, Intramedullary rodding, Ilizarov’s fixator, Union rate, Residual deformities

## Abstract

**Background:**

The purpose of this study was to investigate the initial union rate, refracture rate and residual deformities of congenital pseudarthrosis of the tibia (CPT), using combined surgery including pseudarthrosis resection, intramedullary rodding, autogenous iliac bone grafting and Ilizarov’s fixator, with a mean 5.2 years follow-up.

**Methods:**

We retrospectively reviewed the records and diagrams of patients with Crawford type IV congenital pseudarthrosis of the tibia between February 2007 and March 2010. Patients managed by pseudarthrosis resection, intramedullary rod of the tibia, wrapping autogenous iliac bone grafting and Ilizarov’s fixator were enrolled. We evaluated the bone union rate, tibial alignment, limb length discrepancy (LLD), valgus deformity of the ankle and the frequencies of refracture during period of follow-up.

**Results:**

There were 56 cases enrolled in the study, with a mean follow-up 5.2 years (range, 3 to 6.7 years). The mean age of the patients at surgery was 3.5 years (range, 1.5 to 12.4 years). Fifty (89.2 %) of the 56 patients had primary bone union at site of pseudarthrosis, while 5 obtained union after second surgery and 1 failed. The average time spent to obtain pseudarthrosis initial union was 4.5 months (range, 3.0 to 10.0 months) and mean duration of Ilizarov treatment was 4.7 months (range, 3.2 to 10.4 months). Eleven (19.6 %) patients had proximal tibial valgus with a mean angle of 9.5° (range, 5 to 24°), while 10 (17.9 %) patients had ankle valgus deformities with a mean of 12.3° (range, 6 to 21°). Sixteen (28.6 %) patients had an average 2.2 cm LLD (range, 1.5–4.2 cm). Of the 50 cases who obtained initial bone union of pseudarthrosis, 13 (26.0 %) had refracture which need cast immobilization or secondary surgery.

**Conclusions:**

This combined surgery obtained initial union rate of 89.2 % at primary surgery while the refracture rate is 26.0 %. However, residual deformities such as proximal tibial valgus, LLD and ankle valgus were also existed which should be pay more attention to and dealt with.

**Trial registration:**

This study was registered in ClinicalTrials.gov under the name “The Effect of Combined Surgery in Management of Congenital Pseudarthrosis of Tibia” (NCT02640040), which was released on August 31, 2015.

## Background

Congenital pseudarthrosis of the tibia (CPT) is a rare disorder and characterized by segmental osseous dysplasia and progression to fracture at walking age with a substantial risk of non-union, leg length discrepancy (LLD), and malalignment of the tibia. CPT is recalcitrant to standard treatment and patients with this disorder often require multiple surgical procedures in an effort to achieve bone union and a functional extremity [1–3]. Currently, three surgical techniques have been most commonly used, including vascularized fibular graft, intramedullary rod stabilization associated autogenous bone-grafting or bone morphogenetic protein-2, and Ilizarov technique, obtaining markedly improved primary union rate of CPT (range, 28 to 92 %) [4–7]. With vascularized fibular graft, pseudarthrosis union can be achieved in most patients; however the microsurgery techniques has a long learning curve. Intramedullary rod has been widely used for treatment of CPT to obtain union and protect against refracture, but it is an unstable fixation of the distal tibial fragment, and may cause ankle stiffness and arthritic changes in the ankle joint [8–10]. The Ilizarov technique has a high rate of fusion, allowing for the correction of axial malalignment and leg shortening at the same procedure. However, there is a risk of refracture after removal of the fixator [10–12].

Most recently, a tendency to treat CPT using combined Ilizarov’s technique and intramedullary rod were evolved as being more reliable in achieving and maintaining union, diminishing residual deformities such as valgus angulations, leg-length discrepancy, and refracture [13–15]. Since February 2007, we used a combined surgical technique, including pseudarthrosis resection, intramedullary rod of the tibia, wrapping autogenous iliac bone graft and Ilizarov’s technique for the treatment of congenital pseudarthrosis of the tibia, to manage Crawford IV congenital pseudarthrosis of the tibia.

The purpose of this study was to present our result of combined surgical technique in management of congenital pseudarthrosis of the tibia and investigate the frequency of re-fracture and residual deformities during a mean 5.2 years follow-up.

## Methods

Between February 2007 and March 2010, 56 consecutive cases with Crawford type IV congenital pseudarthrosis of the tibia managed by pseudarthrosis resection, intramedullary rod of the tibia, wrapping autogenous iliac bone grafting and Ilizarov’s fixator, were retrospectively reviewed.

There were 43 boys and 13 girls with the right leg involved in 30 patients, left in 26. Pseudarthrosis was at the lower third of the tibia in 41 patients, middle third of diaphysis in 12 patients, and upper third in the other 3 patients. Forty-eight patients (85.7 %) presented with neurofibromatosis type 1 (NF1) and 8 patients (14.3 %) with unknown etiology. Forty-one patients (73.2 %) had no previous surgery, while 15 patients (26.8 %) underwent 1 to 4 unsuccessful procedures in other hospital prior to referral to our institution. The mean age at the initial operation was 3.5 years (range, 1.5 to 12.4 years). The average limb-length discrepancy (LLD) pre-operatively was 1.6 cm (range, 1.0 to 3.8 cm).

### Surgical technique

#### Harvesting autogenic iliac bone

**Fig. 1:**
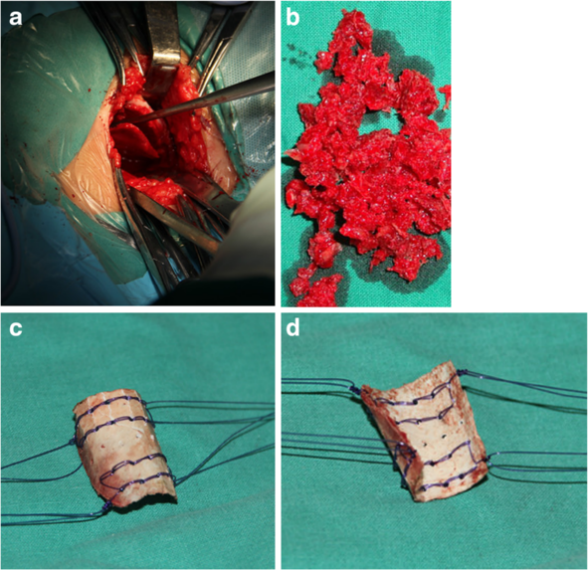
Harvesting and suturing autogenic iliac bone. Exposure of the outer table of the ilium, harvesting a rectangular cortex (**a**). Cancellous bone curetted from supra-acetabular region (**b**). Holes were made in the rectangular cortex with Kirschner wire and with doubled absorbable sutures on each corner (**c**). The rectangular cortex was bent to produce a cylindrical shape for wrapping the cancellous bone graft. It would have greenstick fracture on the cortex (**d**)

The patient was placed in a supine position on an operating table. The iliac bone graft was harvested through a straight incision centered over the anterior superior iliac spine. The apophysis of the ilium was split and the outer table of the anterolateral surface of the ilium was exposed subperiosteally. A rectangular cortex was obtained from the outer table of the ilium and as much cancellous bone as possible was curetted from the supra-acetabular region, while keeping the inner wall intact. Several holes were made in the rectangular cortex with 1.5 mm Kirschner wire and then it was woven sutured with absorbable sutures on each corner and bent to cylindrical shaped (Fig. 1).

#### Excision of pseudarthrosis, intramedullary rod insertion, and installation of Ilizarov’s fixator

A tourniquet was used to decrease the blood loss. An anterior straight incision over the site of the pseudarthrosis was made to expose the pseudarthrosis. The deep fascia of the anterior compartment was routinely divided longitudinally to prevent compartment syndrome. Excision of the pseudarthrosis included the abnormal periosteum, surrounding pathologic soft tissues and the sclerotic bone ends until normal tissue planes are encountered. The medullary canal of both proximal and distal tibial fragment was opened with sequentially larger drill until a 4 mm or 4.5 mm intramedullary rod could be inserted. In patients with concomitant fibular pseudarthrosis, fibrous tissue at site of pseudarthrosis of the fibula was also resected and typically fixed with a 1.6–1.8 mm intramedullary Kirschner wire based on the age of patient and the diameter of the fibula. But in patients with intact fibular, because an intact fibula will hinder the apposition of the tibial fragments, osteotomy of the proximal fibula was performed to allow complete apposition of the ends of the tibia, but the fibula osteotomy was not fixated but to leave both segments overlapped.

A Williams’ rod of appropriated length and diameter determined on digital radiography preoperatively was inserted into the medullary canal of the tibia across the CPT site, from proximal to distal direction via the calcaneus and talus and out of the sole through the heel pad. The fluoroscopy was used to ensure that the rod was located in the center of distal tibial physis on anteroposterior and lateral views and that a neutral dorsiflexion-plantar flexion of the foot and neutral varus-valgus alignment of the ankle were maintained. The rod was then driven retrograde into the proximal tibial fragment, which was anatomically aligned in both the coronal and the sagittal planes was verified using intraoperative imaging.

**Fig. 2:**
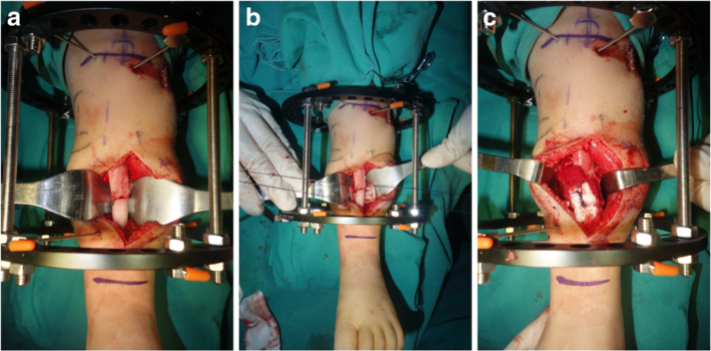
Installation of Ilizarov’s fixator and wrapping bone grafting. Resection of pseudarthrosis and installation of Ilizarov fixator (**a**). Cancellous bone compacted circumferentially between the cylindrical cortex and the pseudarthrosis of the tibia (**b**). The wrapping bone graft was secured by tying the sutures (**c**)

After finishing the insertion of the intramedullary rod, the Ilizarov’s fixator was mounted with one full ring above the site of pseudarthrosis and one below. Both rings were fixed with two or three 1.5 or 2.0 mm tensioned Kirschner wires through the tibia and were connected together by threaded rods, subsequently applying appropriate pressure at site of pseudarthrosis of the tibia. If the distal tibial segment was less than 3 cm long, a calcaneal half-ring or U-shaped ring was used to increase distal stability. In addition, if the tibial length discrepancy was more than 3 cm compared with the normal side of the tibia and the age of patient was more than 3 years, proximal tibial lengthening was performed concomitantly through a proximal metaphysic corticotomy by distraction osteogenesis at initial operation. Another ring was added proximal to the metaphysic corticotomy site. The bone segments proximal and distal to the corticotomy site were fixed with three 1.5 or 2.0 mm Kirschner wires connected to the ring (Fig. 2a).

#### Wrapping autogenic iliac bone graft

The previously harvested the cylindrical cortex was wrapped around site of pseudarthrosis of the tibia after the application of the Ilizarov fixator. The cancellous bone grafts were placed circumferentially between the grafted cortex and site of pseudarthrosis and the cortex was tied with absorbable sutures, which had been connected to the cortex previously, establishing a sealing environment for enhancement of osteogenesis (Fig. 2b and c).

#### Postoperative management

The wound was sutured routinely with retention of a drainage tube. The drainage tube was usually removed within 72 h after surgery. For cases with proximal tibial lengthening, distraction osteogenesis was started 1 week post-operation, and the speed for bone transportation was 0.5 mm/day in 4 increments [13]. The patients were followed every 4 weeks for the first 3 months and every 6 weeks for the next 3 months until radiographic union was obtained. When the pseudarthrosis of the tibia had consolidated, the Ilizarov’s fixator was removed and a short leg cast was applied for an average of 2 months. After the cast is removed, the patient was then fit with a protective knee-ankle-foot orthosis and was allowed to begin weight-bearing. Hereafter, patients were followed at interval of 3 months until the patient reaches skeletal maturity.

In order to minimize the duration of ankle joint immobilization and the potential damage of the articular cartilage with prolonged ankle transfixation of the intramedullary rod of the tibia, the rod across the ankle joint was surgically pushed into epiphysis of the distal tibia at 18 to 24 months after union of pseudarthrosis, using a 2.5 mm K-wire or another rod with threaded end to reach or connect the hollow threaded end of Williams’ rod, and drive the rod into the epiphysis of the distal tibia under fluoroscopy.

#### Evaluation

Sequential radiographs taken during follow-up and clinical chart of each patient were reviewed to evaluate outcome in patient with congenital pseudarthrosis of the tibia treated by this combined surgical technique, including time taken for union at the site of pseudarthrosis, tibial alignment, limb length discrepancy, the status of fibula, and frequency of re-fracture during duration of follow-up.

Union of congenital pseudarthrosis of the tibia was defined as evidence of bridging callus on four cortices across the transverse tibial cortical defects without visible fracture line on both the anteroposterior and lateral radiographs [16].

**Fig. 3:**
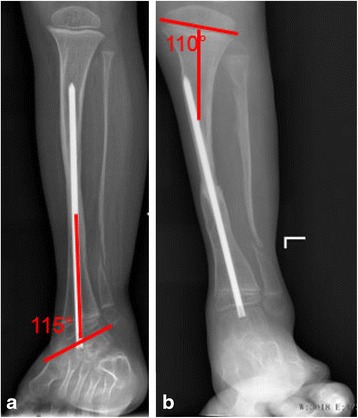
The evaluation on X-ray. Illustrations of the measurements of ankle valgus (**a**) and proximal tibial valgus (**b**) respectively

The assessment of tibial alignment consisted of measurements of the proximal tibial valgus and ankle valgus on anterioposterior radiograph. The measurement of proximal tibial valgus was created by the intersection of a line parallel to proximal physis and another line along the axis of proximal third of diaphyseal in the tibia. Ankle valgus was assessed by tibiotalar angle that was measured at the intersection of the mid-diaphyseal line of the tibia and a line drawn across the flat subchondral line of the talar dome (Fig. 3). If patient who had >3° of proximal tibial valgus and >5° of ankle valgus was defined as proximal tibial valgus and ankle valgus deformity respectively.

The length of involved tibia and contralateral tibia were measured directly from proximal physis to distal physis of tibia on digital X-ray imaging.

Refracture was defined as an obvious fracture on a plain radiograph following the primary union at site of pseudarthrosis.

## Results

There were 56 patients with congenital pseudarthrosis of the tibia treated by our combined procedure enrolled. The mean follow-up was 5.2 years (range, 3 to 6.7 years). Fifty (89.2 %) of the 56 patients had initial union of pseudarthrosis of the tibia. There were 6 cases that did not obtained initial union. Among the 6 cases, 3 achieved union after another attempt of wrapping autogenic iliac bone graft and Ilizarov fixator compression, 2 obtained final union using the Masquelet technique, only one failed union after second surgery (Masquelet technique) and refused to subsequent treatment. The average time spent to obtain initial union was 4.5 months (range, 3.0 to 10.0 months) and mean duration of Ilizarov treatment was 4.7 months (range, 3.2 to 10.4 months).

At recent follow-up, 12 patients had the rod being located in the epiphysis of the distal tibia with growth of the tibia, 4 patients with the rod in the tibia medullary cavity, while the other 40 patients had underwent a surgery to push the intramedullary rod to the epiphysis of the distal tibia.

Eleven (19.6 %) patients had proximal tibial valgus with a mean angle of 9.5° (range, 5 to 24°). Ten (17.9 %) patients had ankle valgus deformities with a mean of 12.3° (range, 6 to 21°). Sixteen (28.6 %) patients had an average 2.2 cm LLD (range, 1.5–4.2 cm).

Of the 50 cases who obtained initial bone union of pseudarthrosis, 13 (26.0 %) had refracture which need cast immobilization or secondary surgery. Ten patients sustained re-fracture at the original pseudarthrosis site, 2 patients at a more proximal site, and one patient at a more distal site. Patients with re-fracture were treated with cast immobilization firstly. If union was not obtained, surgical management was considered. Among the 13 patients with refracture, 8 patients healed at refracture site by cast immobilization, 3 patients obtained solild union by Ilizarov fixator to compress at the refracture site, but the remaining 2 patients failed to retrieve bony union with Ilizarov compression.

Ankle joint stiffness was identified in 4 patients due to the previous failed surgery. There were 11 cases with pin-tract infection which was cured by pin-tract nurse and oral administration of antibiotics. Two cases developed central epiphyso-metaphyseal bone bridge formation, however no growth disturbance was found. There were no other complications such as knee contracture, neurovascular compromise, peroneal nerve injury, or compartment syndrome in any patient.

**Fig. 4:**
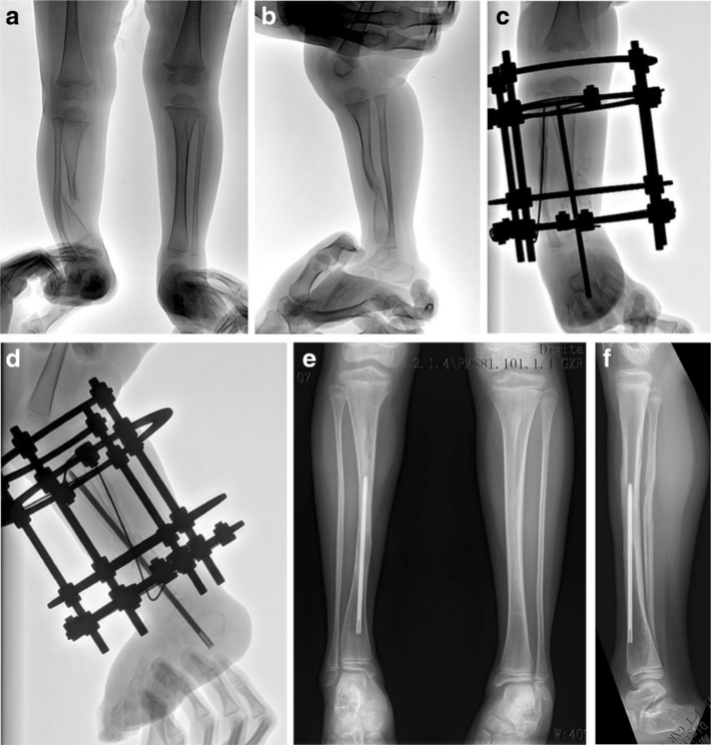
A typical case of 2.5 years boy with congenital pseudarthrosis of the tibia. Preoperative anteroposterior and lateral X-rays shows Crawford type IV CPT and intact fibula (**a**, **b**). Anteroposterior and lateral radiograph of the same patient taken at 2 months after combined surgey (**c**, **d**). X-ray of 5.4 years post operation shows solid union of the pseudarthrosis with good alignment of the tibia without ankle valgus, tibia angulation or LLD. The rod was in the tibia medullary cavity with growth without surgical pushing of the rod (**e**, **f**)

The demographic data of results were summarized in Table 1. We present a typical case in Fig. 4.

**Table 1:** Patients result

Parameters	
Mean age at index surgery	3.5 years (range, 1.5 to 12.4 years)
Follow up	5.2 years (range, 3 to 6.7 years)
Bone initial union time	50 cases (89.2 %)
	4.5 months (range, 3.0 to 10.0 months)
Proximal tibial valgus	11 cases (19.6 %)
	9.5° (range, 5 to 24°)
Ankle valgus	10 cases (17.9 %)
	12.3° (range, 6 to 21°)
Limb-length discrepancy	16 cases (28.6 %)
	2.2 cm (range, 1.5–4.2 cm)
Refracture	13 cases (26.0 %)

## Discussion

Although the outcomes from numerous treatment methods for congenital pseudarthrosis of the tibia had shown greatly improvement in primary union [1, 5], there are still many challenging problems that include potential risks of non-union, re-fractures, leg length discrepancy (LLD), and malalignment of the tibia and the ankle [17]. Currently, there is no consensus on the optimal surgical technique for treatment of congenital pseudarthrosis of the tibia.

Ilizarov technique was widely used to manage with CPT, with the biggest advantage of the tibial fragments being compressed, lengthening of the tibia, and correction of angular deformities concomitantly. The drawback of Ilizarov technique was its lack of protection against re-fractures after removal of the fixator and usually associated with a high re-fracture rate [4, 12]. Inan et al. reported 16 patients treated with Ilizarov, all the patients united but 10 patients had late axial malalignment and re-fracture [17]. Cho et al. reported a rate of re-fracture of 47 % in a series of 23 patients with atrophic CPT treated by Ilizarov’s technique [18].

Intramedullary rod was another commonly method used for treatment of CPT popularized by Umber et al. in 1982 and obtained relative high union rate [5, 19]. An additional benefit of this technique could provide protection against re-fracture before skeletal maturity [1, 20]. Dobbs successfully treated 18 of 21 patients with intramedullary rod but re-fracture occurred in twelve patients [9]. Joseph and Mathew treated 14 patients with congenital pseudarthrosis of the tibia using excision of the pseudarthrosis, double onlay autogenous cortical bone grafting, and intramedullary nailing [21]. Of 14 pseudarthrosis of the tibia, 12 patients achieved union without re-fractures occurred at the site of the original pseudarthrosis. But in practice, we found the onlay cortical bone grafting to be unstable and may be absorbed spontaneously during the fixation. Thus, since February 2007, we used intramedullary rod and wrapping autogenic iliac bone graft in combination with Ilizarov’s fixator for treatment of congenital pseudarthrosis of the tibia and attempted to take advantages of both Ilizarov’s fixator and intramedullary fixation, and avoid the disadvantage of onlay cortical bone grafting. The Ilizarov’s fixator could provide high fusion rate with good alignment control, while intramedullary fixation could stabilize the pseudarthrosis and avoid re-fracture. The wrapping autogenous cortex and cancellous bone grafting would make the grafting and pseudarthrosis close contacted over a large surface area, which may enhance mechanical stability and provide a unique biologic environment promoting union.

In current study, 56 patients with congenital pseudarthrosis of the tibia were treated by the combined procedure and 50 (89.3 %) patients achieved initial union at site of pseudarthrosis at a mean follow-up of 5.2 years, while another 5 case obtained union after second surgery, only 1 failed union. During a 5.2 years follow up (the shortest was 3.5 years), we observed the total refracture rate was 26.0 %. The primary union rate and the refracture rate of ours were comparable to those reported in the literature [8, 21, 22]. However, high frequency of residual deformities including proximal tibial valgus, ankle valgus, LLD and still existed, which is similar to Dobbs and Kristiansen’s study [9, 12].

The incidence of proximal tibial valgus post opreration was 19.6 % with an average of 9.5°, and we observed proximal tibial dysplasia in these patients. Cho et al. pointed that the proximal tibial dysplasia reflects a pathological process of the periosteum same as fibrous hamartoma in the pseudarthrosis site, and the extent of this lesion varies in patients [23]. We surmise that proximal tibial dysplasia may result to unbalanced growth of proximal tibial which seems to be attributable to tibial valgus of our series. Epiphysiodesis by staple or screw fixation can be introduced to correct tibial valgus in these younger patients.

Ten (17.9 %) patients had ankle valgus deformities post operation with a mean of 12.3°, which is similar to other investigators [9, 10]. Some authors reported that persistent fibular pseudarthrosis may relate to progressive ankle valgus [12, 17]. Distal tibia corrective osteotomy, fibular stabilization by temporary hemiepiphysiodesis or tibiofibular synostosis would be advocated if serious ankle valgus deformities were existed.

An average of 2.2 cm LLD in 16/56 case was observed in our series. The LLD following success union may ascribe to the discrepancy of limb length pre-operation and the acute shortening at the time of the pseudarthrosis resection. Either epiphysiodesis of the contralateral side or tibial lengthening of the involved side could be used to equalize the limb length [12, 17]. In our experience, we suggested that proximal tibial lengthening could be considered when the LLD was more than 4 cm in younger children after primary union of CPT [13].

Residual problems such as proximal tibia valgus, ankle valgus, LLD and refracture are common despite successful union of pseudoarthrosis, thus more attention should be paid to these residual deformities and revision surgeries may need to correct these deformities.

The present study has three limitations. First, it was not long enough to document real incidence of residual deformities with this combined approach, including proximal tibial valgus, ankle valgus, LLD, and refracture. It is necessary to follow up these younger patients until skeletal maturity and to evaluate the long term outcome of the combined technique. Second, we need to carry out some well designed prospective studies to investigate the advantage of wrapping autogenic iliac bone graft. Last but not least, due to its retrospective properties, some data were not available to carry out comparative research for this series.

## Conclusion

This combined technique has advantages of both allowing tibial fragments being compressed at site of pseudarthrosis of the tibia to facilitate bony union and providing protection against re-fracture. However, residual deformities such as proximal tibial valgus, LLD and ankle valgus were also existed which should be pay more attention to and dealt with. It is necessary to follow up until skeletal maturity and to evaluate the long term outcome of the combined technique.

## Abbreviations

CPT: Congenital pseudarthrosis of the tibia
LLD: Limb length discrepancy
NF1: neurofibromatosis type 1
